# Generative artificial intelligence in health technology assessment: a patient and citizen dialogue on trust, human oversight, and shared governance

**DOI:** 10.1017/S0266462326103560

**Published:** 2026-02-23

**Authors:** Fiona Pearce, Aline Silveira Silva

**Affiliations:** HTAi Patient and Citizen Involvement in HTA Interest Group, Canada

**Keywords:** generative artificial intelligence, patient and citizen involvement, technology assessment, health

## Introduction

The integration of artificial intelligence (AI), particularly generative artificial intelligence (GenAI), into health technology assessment (HTA) activities is progressing rapidly. The 2025 Health Technology Assessment international (HTAi) Global Policy Forum (GPF) provided a timely and comprehensive examination of opportunities, challenges, and governance considerations associated with this shift. Their resulting publication (1) reflects a strong sense of momentum, with participants broadly aligned on the transformative potential of GenAI to improve efficiency, analytical capacity, accuracy, and timeliness within HTA processes, while also acknowledging the ethical, social, and institutional challenges that must be addressed to support its responsible adoption.

From the perspective of the HTAi Patient and Citizen Involvement in HTA Interest Group (PCIG), this moment represents both an opportunity and a point of caution. AI is not simply another methodological innovation within HTA; it is a systemic change that has the potential to reshape how evidence is generated, interpreted, and deliberated. As such, it raises fundamental questions about whose knowledge counts, data privacy, ethical use, how legitimacy is sustained, and what it means to involve patients and citizens in decision-making processes increasingly supported by algorithmic tools.

This Dialogue article builds on the GPF publication by exploring what responsible AI adoption in HTA looks like when viewed through a patient- and citizen-centered lens. Rather than offering prescriptive solutions, it explores how the use of GenAI in HTA may either support or undermine human decision-making depending on how it is governed and implemented, and seeks to stimulate further discussion on how patient and citizen involvement must evolve in parallel with technological change as AI moves from exploration to routine use.

## Trust in AI: a technical problem or a social one?

Trust emerged as a central theme during the GPF discussions, with transparency, validation, auditability, and disclosure of AI use identified as key enablers of confidence in GenAI tools. In addition, the GPF highlighted that trust may be undermined by broader ethical and social concerns related to AI use, including patient data privacy and the environmental impact of AI systems. From a patient and citizen perspective, these concerns are closely linked to questions of consent, data stewardship, and social responsibility, as well as to whether the efficiency gains promised by AI justify its wider societal and environmental costs.

However, trust in AI-supported HTA is not solely a technical challenge; it is also a social and relational one. Patients and citizens already often feel that they are not sufficiently included in HTA processes and are uncertain about how their contributions are used or whether they influence outcomes. The introduction of GenAI risks reinforcing these perceptions if AI is seen as another layer separating lived experience from decision-making.

When applied thoughtfully and responsibly, GenAI may offer opportunities to strengthen patient and citizen involvement in HTA. Techniques such as large-scale data analytics and natural language processing (NLP) can support the systematic aggregation and analysis of qualitative inputs, including patient narratives, open-ended survey responses, and public submissions, helping to ensure that lived experiences are reflected more consistently and transparently within HTA processes. However, these potential benefits must be accompanied by robust safeguards to protect data privacy, and clear governance arrangements regarding data use and ownership. In parallel, the environmental footprint of AI-supported approaches should be considered, with proportional and sustainable use needed to ensure that efforts to enhance operational efficiencies do not introduce avoidable ethical or societal harms.

The *PCIG Values and Quality Standards for Patient Involvement in HTA* (2) position transparency not only as disclosure, but as a means of enabling understanding and dialogue. Applied to GenAI, this suggests that transparency must include clear communication about why AI is being used, what problems it is intended to address, and its limitations. Importantly, it also requires openness about uncertainty and error, particularly where AI outputs may influence deliberations that affect access to care.

Patients and the public are also increasingly aware that AI systems can replicate existing biases, obscure accountability, and operate in ways that are difficult to explain. While technical transparency, such as documenting data sources, model choice, and human oversight is necessary, it may be insufficient on its own to build public trust. There is a risk that AI governance in HTA becomes overly procedural, focusing on compliance checklists rather than on legitimacy in the eyes of those affected by decisions.

In this context, accountability is a critical complement to transparency. Clear lines of responsibility must be maintained to ensure that the use of AI does not diffuse accountability or make decisions harder to scrutinize or challenge. For patients and citizens, knowing who is accountable for AI-supported decisions, and how concerns can be raised and addressed, is fundamental to maintaining trust.

Trust, therefore, is shaped not only by how AI performs but also by whether people feel their values, lived experiences, and concerns have influenced how AI is designed and used. HTA systems will struggle to gain acceptance if people do not understand how AI outputs are integrated into decision-making, where accountability lies, or whether patient and citizen perspectives have informed system design and application.

If these conditions are not met, AI-supported HTA may gradually lose relevance. Legitimacy in HTA comes not just from technical rigor, but from reflecting societal values and real-world needs. Systems that prioritize algorithmic efficiency over deliberation, inclusion, and accountability may become disconnected from the populations they are intended to serve.

Trust is further influenced by the depth and timing of patient and citizen involvement. Engagement that occurs only after AI tools are embedded in routine practice risks being perceived as tokenistic. By contrast, involving patients early in discussions about acceptable use cases, data governance, and risk tolerance can contribute to a sense of shared ownership, which is essential for building and maintaining trust.

## Human oversight of the use of GenAI

The GPF strongly emphasized the importance of ensuring AI augments, rather than replaces, human decision-making, using the concept of “human-in-the-loop” (HITL) positioned as a key safeguard against inappropriate automation. Central to these discussions was the need to maintain human agency (defined as the ability for humans to retain meaningful control over, and influence on, AI systems and their outputs).

From a PCIG perspective, this emphasis is welcome but insufficient on its own. The critical question is not simply whether humans remain in the loop, but which humans are involved, and in what capacity. HITL frameworks often prioritize expert oversight, ensuring that HTA analysts, reviewers, or committee members validate AI outputs and retain final decision-making authority. While this is crucial, there is a risk that patient and citizen perspectives could be sidelined if AI-generated summaries, models, or briefing materials implicitly prioritize certain types of data or narratives.

PCIG recognizes experiential knowledge as a critical component of evidence and maintains that patient and citizen contributions should meaningfully influence HTA outcomes. Applied to AI, this raises important questions: How are patient and citizen perspectives represented in AI-supported processes? Who determines how patient-and citizen-derived data are interpreted or summarized by GenAI tools? And how can we ensure that automation does not inadvertently marginalize qualitative, experiential, or contextual forms of evidence?

Human agency, therefore, should be understood as plural rather than singular. It includes not only expert judgement but also the ability of patients and citizens to shape how evidence is framed, what questions are asked, and how uncertainty is interpreted within HTA deliberations. Without this broader conception, HITL risks becoming a procedural safeguard or technical reassurance, rather than a meaningful ethical commitment.

## Risk-based approaches: Whose risk counts?

The GPF proposed risk-based approaches to GenAI use in HTA, recognizing that different applications carry varying levels and types of potential harm. This pragmatic framing is appealing, yet it also raises important questions about whose perspectives inform how risk is defined, assessed, and managed.

Technical and institutional risks, such as errors in evidence synthesis or inappropriate influence on decision-making, often dominate these discussions. Patients and citizens, however, may prioritize different concerns, including risks related to fairness, transparency, loss of voice, or erosion of trust in government and public institutions. There is a danger that risk-based frameworks developed without patient input may underestimate or overlook these social and ethical dimensions. For example, a GenAI application deemed “low risk” from an operational standpoint, such as summarizing patient submissions, may nevertheless have significant social or ethical implications if it alters how lived experience is represented, interpreted, or weighted within assessments.

Co-developing risk frameworks with patients and citizens can help surface these concerns early and reduce the likelihood of unintended consequences. It may also support more consistent adoption across jurisdictions by grounding governance approaches in shared values and lived experience, rather than relying solely on technical thresholds or institutional risk classifications.

## Equity and capacity: Who benefits from AI in HTA and who is left behind?

The GPF acknowledged that AI adoption in HTA is unfolding unevenly across regions, with most activity currently concentrated in jurisdictions with mature HTA systems and greater technical capacity. From a patient and citizen perspective, this unevenness raises concerns about widening global inequities. In settings with limited HTA capacity, GenAI tools could offer important benefits by supporting evidence synthesis, improving efficiency, and reducing resource constraints. However, without adequate governance, capacity-building, and social engagement, these tools may also introduce new dependencies or exacerbate existing inequities. Patients and citizens in low- and middle-income countries may have limited opportunities to influence how AI is adopted and governed, despite AI-supported decisions having direct implications for access to care.

Equity, therefore, should be considered not only in terms of access to AI tools, but also in terms of voice and influence over how those tools are designed, implemented, and used. Global HTA collaboration on AI must include mechanisms to elevate patient perspectives from diverse settings, support context-sensitive approaches to governance, and avoid exporting models that may be poorly aligned with local health system priorities, values, or capacities.

## Questions from the PCIG community

As GenAI moves from exploration to implementation in HTA, several questions emerge from a patient and citizen perspective:

- How can patient and citizen involvement be systematically integrated into AI governance structures, rather than treated as a downstream or consultative add-on?
- What safeguards are needed to ensure that AI-supported efficiency gains do not come at the expense of deliberation, inclusiveness, or legitimacy in HTA decision-making?
- How should accountability, data privacy, transparency, and environmental sustainability be governed in the use of AI within HTA?
- How should HTA bodies communicate the use of AI to the public in ways that are meaningful, accessible, and build trust?
- What responsibilities do HTA networks and international collaborations have to promote ethical, sustainable, equitable and participatory AI adoption across diverse health systems and contexts?

Addressing these questions will require continued collaboration across all those involved in HTA, alongside a willingness to treat AI not only as a technical innovation, but as a catalyst for re-examining how values, evidence, and voices are balanced within HTA. For the PCIG community, this reflection is essential to ensuring that the integration of GenAI strengthens, rather than weakens, the legitimacy and social purpose of HTA.

## Concluding reflections

The HTAi GPF has provided an important foundation for advancing dialogue on the use of GenAI in HTA. From the perspective of PCIG, the next phase of this work must ensure that patient and citizen involvement in AI use evolves alongside technological change, rather than lagging behind it.

As HTA systems adapt to an AI-supported future, inclusive discussions across all individuals and groups involved in HTA processes, including patients and citizens, will be essential. Responsible AI adoption in HTA will depend not only on governance frameworks and risk management strategies, but also on deliberate and sustained efforts to build trust through inclusion, transparency, and shared decision-making. GenAI has the potential to reshape HTA processes profoundly; whether it ultimately strengthens or weakens their legitimacy will depend on how intentionally patient and citizen voices are embedded throughout this transformation.
